# Exploitation Characteristics of Teeth Flanks of Gears Regenerated by Three Hard-Facing Procedures

**DOI:** 10.3390/ma14154203

**Published:** 2021-07-28

**Authors:** Svetislav Marković, Dušan Arsić, Ružica R. Nikolić, Vukić Lazić, Branislav Hadzima, Vladimir P. Milovanović, Renata Dwornicka, Robert Ulewicz

**Affiliations:** 1 Faculty of Technical Sciences Čačak, University of Kragujevac, 32000 Čačak, Serbia; svetom@mts.rs; 2 Faculty of Engineering, University of Kragujevac, 34000 Kragujevac, Serbia; dusan.arsic@fink.rs (D.A.); vlazic@kg.ac.rs (V.L.); vladicka@kg.ac.rs (V.P.M.); 3 Research Center, University of Žilina, 010 26 Žilina, Slovakia; branislav.hadzima@uniza.sk; 4 Faculty of Mechanical Engineering, Cracow University of Technology, 31155 Cracow, Poland; renata.dwornicka@mech.pk.edu.pl; 5 Department of Production Engineering and Safety, Czestochowa University of Technology, 42201 Czestochowa, Poland; robert.ulewicz@pcz.pl

**Keywords:** regeneration, gear, hard-facing, hardness, microstructure, hereditary properties

## Abstract

Numerous phenomena that occur during the process of machine parts’ regeneration have a significant impact on the loss of their working ability. Therefore, the properties of the working surfaces of the teeth flanks of repaired gears were analyzed in this research. The hereditary properties of the gear teeth are expressed by the interdependence of their geometric and physical-mechanical-metallurgical parameters created during the technological operations of regeneration of worn teeth by welding/hard-facing. The hard-facing was executed with three filler metal types, namely: combination Inox 18/8/6 + EDur 600, Castolin 2 and UTP 670. The tested properties included geometrical accuracy, microstructure and microhardness. Evaluation of the executed regeneration procedures was done by comparing the mentioned parameters of the regenerated gears and the new ones. The tested gears were not withdrawn from production due to damage, but they were newly manufactured and intentionally damaged gears, made of the same materials, subjected to the same manufacturing process. In this way, all influences except for the considered filler metal type were eliminated. Based on results of the conducted experiments, it was possible to establish the influence of the filler metal type on the surface characteristics of the regenerated gears’ teeth flanks.

## 1. Introduction

The objective of this paper was to present the dependence of the physical-mechanical-metallurgical parameters of the gear teeth flanks, which are the technological inheritance of the selected filler metals used for their regeneration by hard-facing. In addition to that, an attempt was made to prescribe the optimal technology for the reparatory hard-facing of the gear teeth by the so-called “hard” electrodes. These hard electrodes should be made up of filler metals that provide for the required surface hardness of the teeth without additional thermal-chemical treatment. The way of solving this, by no means an easy task, is illustrated by the presented results of the experimental investigation. To determine the influence of the filler metal on the acquired (inherited) properties of the regenerated gears, it was necessary to conduct voluminous experimental investigations on teeth regenerated by the hard filler metals, Inox 18/8/6 + EDur 600, Castolin 2 and UTP 670, by the application of the two different hard-facing procedures. The tested properties included the geometrical accuracy, microstructure and microhardness of the teeth flanks. Evaluation of the executed regeneration procedures was done by comparing the mentioned parameters of the regenerated gears to the same parameters of the new ones.

Investigations in the area of the regenerated gears, conducted until now, have shown that reparatory hard-facing of damaged working surfaces is the most reliable renewal method of shapes, dimensions and working characteristics of teeth. For the proper selection of an adequate reparatory hard-facing method, it is necessary to know all the mechanisms of wear of surfaces in contact. The regeneration of gears by hard-facing itself is a complex process, consisting of a large number of technological operations, the order of execution of which is extremely important and precisely determined.

Limited literature on the subject has induced the necessity to make the selection of the filler metals based on the authors own experimental experience, as well as that of other researchers. Lazić et al. [1,2] and Arsić et al. [3] have pointed to the possibility of using reparation for restoring the damaged parts of various machine systems. Desir [4] has presented the fact that the Castolin company was confronted with an increasing number of fractures of steel parts; thus they developed the procedures of reparatory welding and hard-facing and presented the metallurgical and technical justification for the presented solutions. Arsić et al. [5] have analyzed the fatigue properties of complex structures and the possibility of their reparation. Borego et al. [6] have analyzed parameters of reparatory welding and hard-facing in order to achieve better fatigue strength of steel for manufacturing casting molds. Atxaga and Irrisari [7] have analyzed causes for engines’ shaft failure due to poorly executed reparation, which they have proved by metallographic investigation, while Czuprynsky [8] has pointed to the fact that the mechanical properties obtained by hard-facing could be similar to the properties of the original material of the repaired part.

Based on analysis of the available literature, one can conclude that a small number of authors have considered the influence of regeneration on the operational characteristics of gears, especially from the aspect of technological heritage. Various hard-facing methods and their application in the reparation process of gears were considered in the authors’ own investigations [9,10,11,12,13], where results of experimental metallurgical investigations of the repaired gear teeth were analyzed. It was concluded that the damaged gear teeth can be repaired by careful selection of the filler metal and welding technology [13,14]. The service life of gears regenerated by hard-facing is close to the service life of the newly manufactured ones. In preparing this manuscript, several other works were studied, which dealt with damage to gears and their restoration for continued use [15,16,17,18,19,20,21,22,23,24,25,26].

## 2. Preparation of Samples for Testing

**Figure 1:**
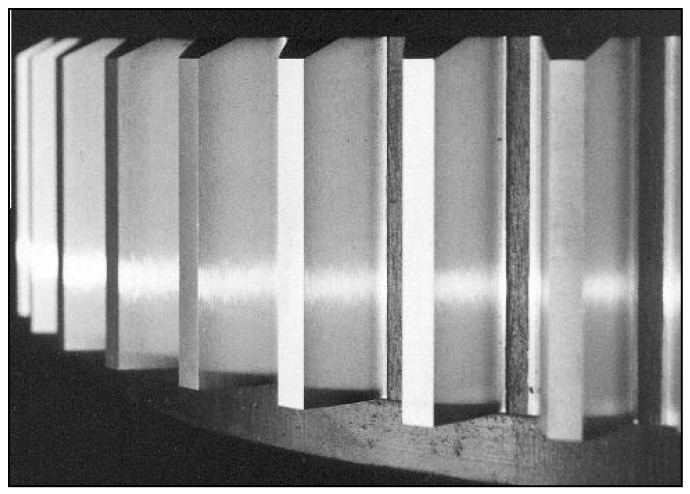
Newly manufactured gear.

This research did not deal with the regeneration of already damaged gears taken out of machine systems that had been in use for a certain period. On the contrary, new gears were made, which were then intentionally damaged and then regenerated. Thus, all the tested gears were made of the same material (steel for cementation, 20MnCr5, whose chemical composition is shown in Table 1) and belonged to one batch. In addition, they were all machined on the same machines with the same machining modes. The gears made this way, Figure 1, had the characteristics presented in Table 2.

Since the chemical composition of steel primarily determines the behavior of austenite during the cooling, it proved necessary to find a simpler, unique system for evaluating the influence of carbon and various alloying elements on stability of austenite and, thus, on the possibility of its transformation into martensite during the welding process. Therefore, the concept of equivalent carbon (*C_E_*) was introduced as an indicator of the metallurgical weldability of steel. The equivalent carbon is calculated by adding the contents of the present alloying elements, multiplied by the appropriate coefficients, to the carbon content of the steel. These correction coefficients represent the influence of the ratio of the individual alloying elements and carbon on a certain property of steel [2].

For the weldability estimate of alloyed steels, the most frequently used expression, recommended by the International Institute of Welding (IIW), is as follows:

(1) $$C_{E}=C+\frac{Mn}{6}+\frac{Ni}{15}+\frac{Cu}{15}+\frac{Cr}{5}+\frac{Mo}{5}+\frac{V}{5}.$$

Steels for which the equivalent carbon value is greater the 0.45% belong to a group of conditionally weldable materials. For the tested steel, that value is within the limits 0.55–0.71%, which is higher than the limit value (0.45%), thus the 20MnCr5 steel is sensitive to the appearance of cracks, and the application of preheating, prior to hard-facing, is necessary. The preheating temperature can be approximately determined according to Seferian’s formula:

(2) $$T_{p}=350\cdot \sqrt{C_{E}-0.25}$$

where, entering the limit values of equivalent carbon, gives the range of values 191–237 °C. The adopted value for these tests was 230 °C to compensate for the heat losses that appear due to carrying the gears from the furnace to the welding stand at the beginning the hard-facing.

Other methods for weldability estimates exist today; however, according to all the criteria, the 20MnCr5 steel for cementation belongs to conditionally weldable materials. Thus, it is necessary to preheat the gear prior to hard-facing, select the adequate welding technology and the hard-facing parameters and apply the post-welding heat treatment for the purpose of reducing the residual stress level. The notation of the applied filler metals, their sizes (diameter, D) and current intensity (I) are given in Table 3, while the chemical composition and the most important mechanical properties are given in Table 4. The applied procedure was manual metal arc welding (MMAW) (111).

The hard-facing method was selected based on the available resources and equipment at the moment, as well as because the MMA welding procedure was proven to be very reliable during the execution of some former reparation processes [2,3,9,26]. Other hard-facing methods, which can be used should also be mentioned, such as GMAW (gas metal arc welding), plasma hard-facing or laser cladding, the application in practice of which is constantly increasing. For instance, if one considers laser hard-facing, it is well known that such a process uses a laser beam to fuse a material with enhanced thermal and mechanical properties on a substrate. The added material is introduced into the process directly, using inert gases, such as argon or helium. The laser cladding can achieve excellent metallurgical bonding between the substrate and the added material, while the cladded layer is relatively pure [27]. Laser hard-facing is employed in the reparation of gears, as well [28,29], and its application also produces good results. The advantage of laser hard-facing lies in the fact that the heat source is narrowly concentrated at a certain point on the material, which results in better accuracy during the hard-facing, as well lesser influence of the heat on the surrounding base metal, which significantly reduces the size of the heat-affected zone and eliminates the appearance of the brittle phases and cracks.

**Figure 2:**
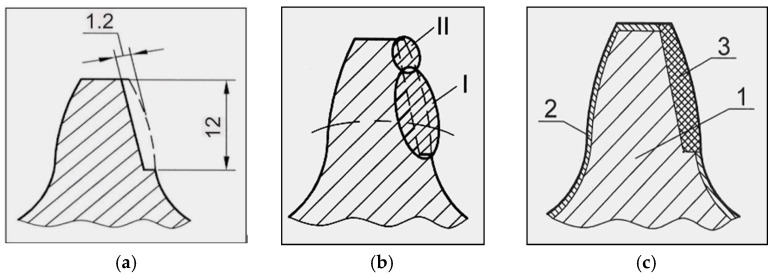
Schematic presentation of the tooth: (**a**) prepared for hard-facing; (**b**) hard-faced in two passes (I—the first pass, II—the second pass); (**c**) cross-section after the hard-facing and machining: 1—tooth core, 2—cemented layer, 3—hard-faced portion.

Technological processes of the gears’ regeneration by reparatory hard-facing with “hard” filler metals are different for the first procedure with respect to the other two. The regeneration process with Inox 18/8/6 and EDur 600 is done by hard-facing in two layers, while hard-facing with the other two types of the filler metals (Castolin 2 and UTP 670) is somewhat simpler and faster, Figure 2a,b. The complete technological process with a detailed description of all the operations for the two types of regenerations is presented in [13].

**Figure 3:**
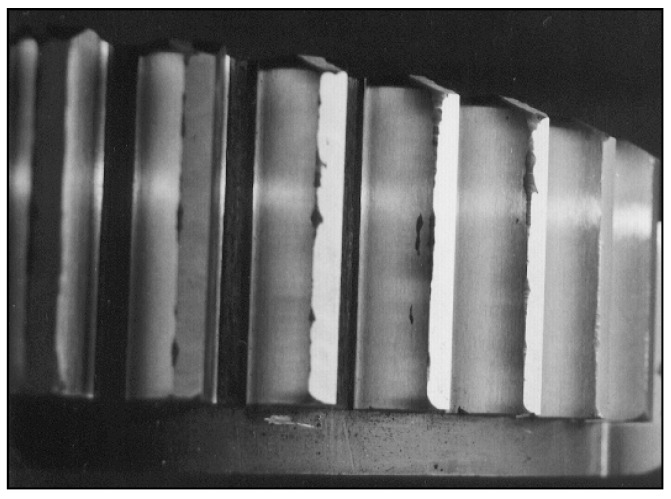
Appearance of the tooth regenerated by hard-facing.

Hard-facing of teeth was done in two passes. In the first pass (I), the tooth base and part of the head were hard-faced, while, after removing the slag, in the second pass (II), the remaining portion of the tooth head was hard-faced. The appearance of the tooth after the hard-facing, heat treatment and machining is shown in Figure 2c. During the hard-facing, it was taken into account that the thickness of the weld must be increased by the size of the additions for the intended subsequent machining. The appearance of the gear regenerated by the hard-facing is shown in Figure 3.

It should be emphasized that the process of this work-piece (tooth) manufacturing was not a simple one, due to the material’s high hardness. That is, after the hard-facing of the gear’s tooth, the problem appeared of the material’s machining and selection of the cutting tool that is capable of providing a tooth surface of the required quality. The problem was solved by the application of super-hard diamond cutting platelets with intensive cooling by a special coolant and lubricant, while some gears were machined directly by profiled whetstone grinding.

## 3. Tribological, Metallurgical and Hardness Investigations

The geometrical measures of samples were controlled by universal and special control/measuring devices. The tooth profile, the tooth flank directions and radial deviation were checked on an involute-meter the “Klingelnberg”, while the regenerated surfaces quality was investigated by the comparative method, i.e., the controlled surface was compared to the corresponding standard with the profile’s medium arithmetic deviation R_a_. Surface hardness was measured on the “Leitz Wetzlar” device by the Rockwell method (HRC). The measured values of characteristic parameters are presented in Table 5 [26].

The tribological investigation was performed on model samples, prepared from the hard-faced surfaces, on a tribometer by the “block-on-disc” method, similar to some previous testing [30,31]. The samples were prepared in such a way to simulate the operation conditions of teeth of the coupled gears. Based on the conducted tribological tests, the topography of the hard-faced surfaces was determined, namely the width of the wear trace was measured on the universal microscope, UIM-21, which is presented in Table 6 [12,26]. The results of calculated friction coefficients are included in Table 6, as well.

From Table 6, one can see that the best quality of surface (the smallest value of the wear trace width and the friction coefficient) was obtained for the hard-facing performed with the combination of the filler metals Inox 18/8/6 + EDur 600, while the worst quality was obtained for the hard-facing with the Castolin 2 filler metal.

Metallographic investigations were conducted on a quantitative optical metallographic microscope of the “Polyvar-Met” type (produced by “Leica Reichert-Jung, Vienna, Austria) at magnifications of 20× to 20,000×. The microstructural analysis was performed on the “Leica Q500MC” device, while the photography and measurements were done in the “QWin” program.

**Figure 4:**
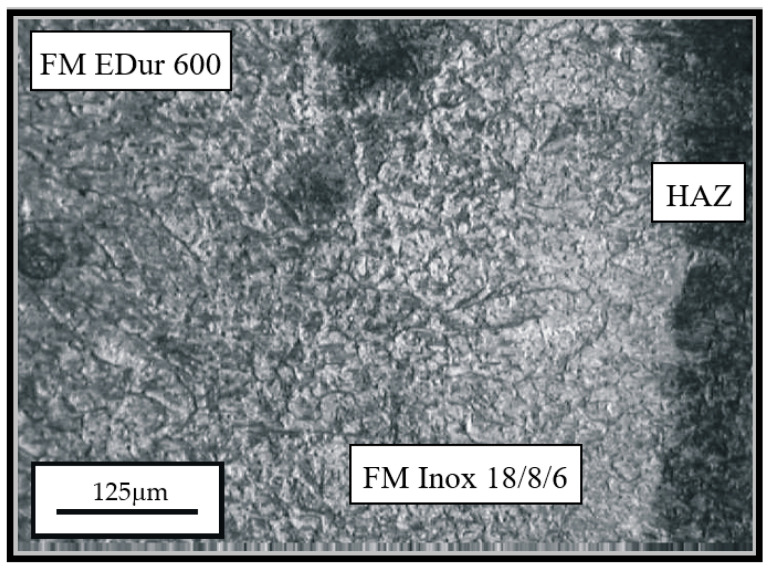
Appearance of microstructure of the hard-faced sample obtained by the two filler metals combination: FM EDur 600—left, FM Inox 18/8/6—middle; HAZ—right.

**Figure 5:**
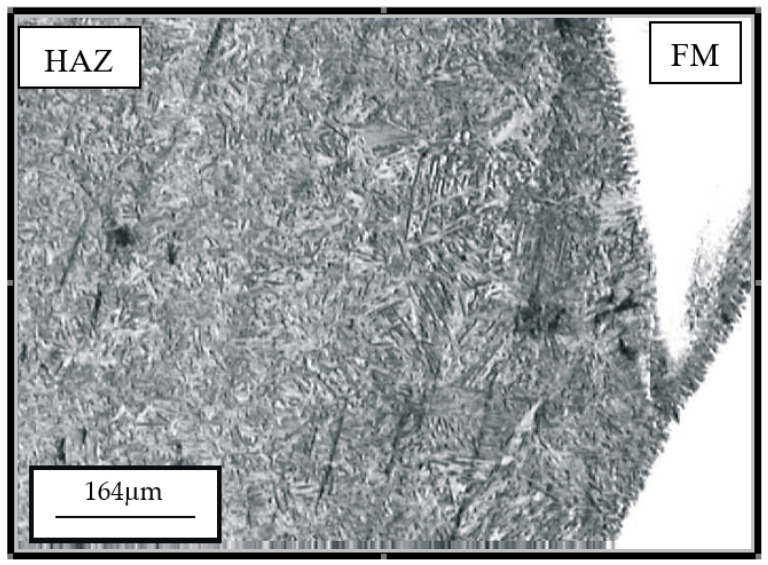
Appearance of microstructure of the hard-faced sample obtained by the two filler metals combination: transition from the HAZ (left) to the FM (right).

The hard-faced layers’ microstructure, obtained by the combination of filler metals (Inox 18/8/6 + EDur 600), is presented in Figure 4 and Figure 5. On the left-hand side of Figure 4, one can notice the needle-like dendritic structure of the hard-faced layer executed by the “hard” electrode EDur 600. In the central area of the figure, one can see the weaker exhibited dendritic structure, which was obtained by hard-facing with the Inox 18/8/6 filler metal, while in the right-hand portion is the heat-affected zone. The joining line between the FM Inox 18/8/6 layer and the HAZ of the base metal is relatively clearly visible, and it is quite uneven. One can notice the mixing of the two materials within the HAZ, Figure 5.

**Figure 6:**
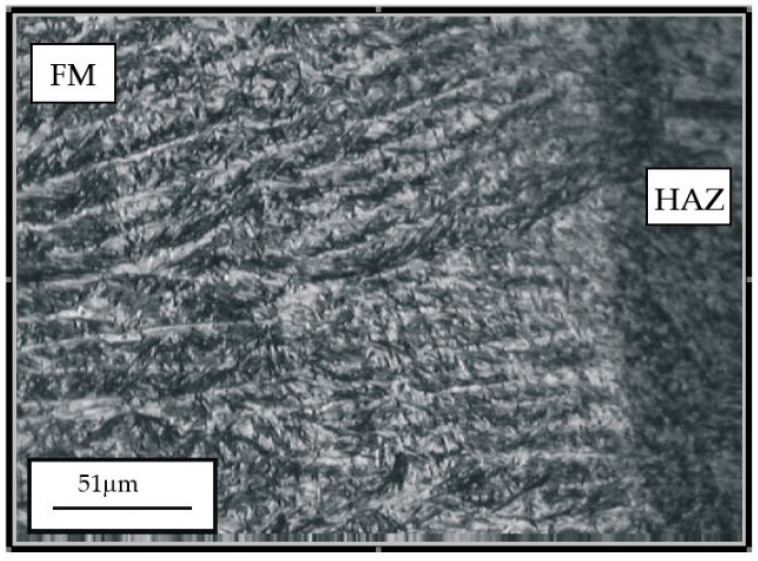
Appearance of microstructure of the hard-faced sample obtained by the Castolin 2 filler metal at the transition between the hard-faced layer and the heat-affected zone.

**Figure 7:**
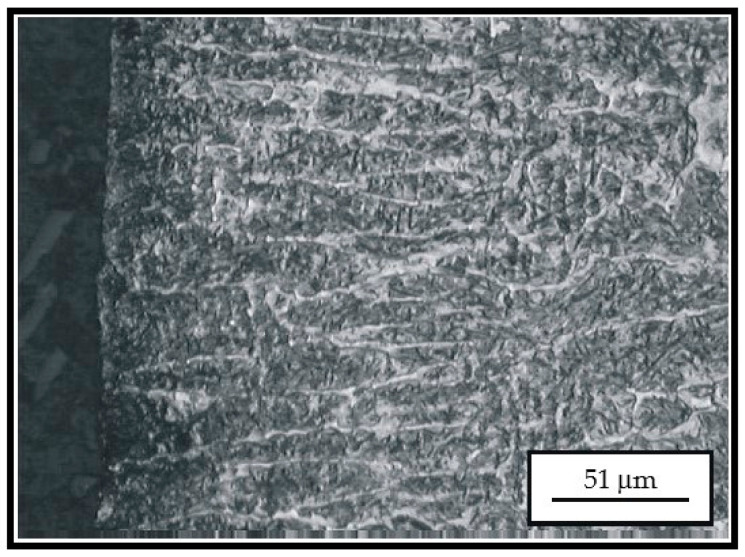
Appearance of microstructure of the hard-faced sample obtained by the Castolin 2 filler metal.

**Figure 8:**
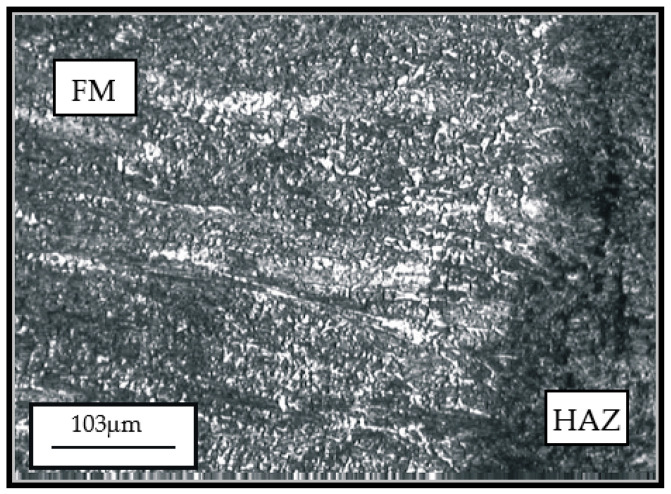
Appearance of microstructure of the hard-faced sample obtained by the UTP 670 (brighter areas) and the heat-affected zone of the base metal (darker areas).

Appearance of microstructure of the hard-faced sample obtained by the Castolin 2 filler metal is presented in Figure 6 and Figure 7, while the microstructure obtained by hard-facing with the UTP 670 filler metal is given in Figure 8.

According to Figure 6 and Figure 7, hard-facing with filler metal Castolin 2 produced a prominent dendritic structure of the hard-faced layer. The average width of the dendritic needles was 10 to 15 μm, which is very convenient. However, the dendrites were characterized by inhomogeneity, which was proven by the various level of surface etching. The carbide phase (primary carbides) was present in the hard-faced layer, as well, mainly extruded along the grain boundaries. The dendrites’ growth direction was mainly perpendicular to the hard-faced layer surface.

The tooth hard-faced with the UTP 670 filler metal possessed a medium-coarse structure with an individual dendrite width of 30 to 80 μm. A high share of the carbide phase was present in the structure, which can be seen as the brighter areas in Figure 8. Carbides were extruded along the grain boundaries; however, the largest portion was in the grains themselves.

Regarding the size and width of the dendrites, the optimal shape of the dendritic phase was obtained by hard-facing with the Castolin 2 filler metal. The microstructure of the surface and subsurface layers of the hard-faced samples obtained by the MMAW welding procedure and the UTP 670 filler metal was somewhat better than for the other filler metals.

The surface hardness was measured on the “Leitz Wetzkal” device by the Rockwell method (HRC) on the hard-faced teeth, at five points. The hardness average values are presented in Table 7. The measured surface hardness values were within the required limits, 58 ± 3 HRC.

**Figure 9:**
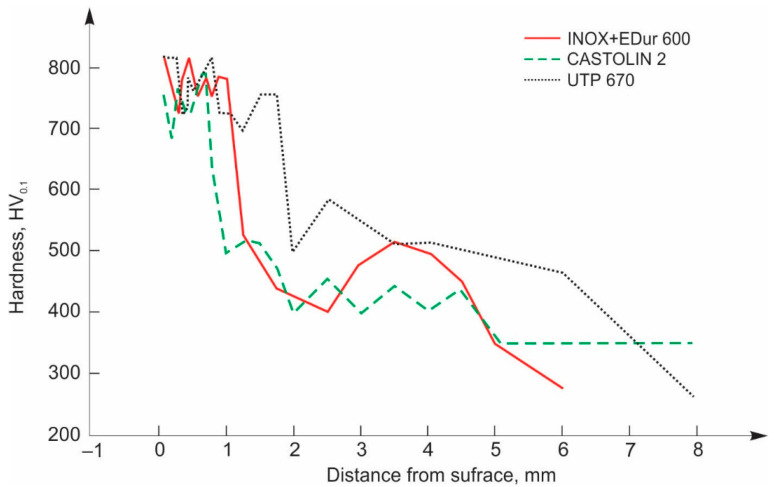
Microhardness distribution of samples regenerated by the hard filler metals.

The microhardness was measured along the cross-section of individual teeth, by the Vickers method (HV_0.1_) with a load force of 1 N and indentation time of 15 s. Results are presented in Table 8, Table 9 and Table 10 and Figure 9.

## 4. Discussion

If one analyzes the hardness distribution of the hard-faced gears, it can be unequivocally confirmed that better properties of hardness in the surface layers of teeth were achieved by application of the hard-facing. The results show that hardness at the teeth surface has high values of about 800 HV, while it decreases toward the bulk of the teeth (Figure 9). At the beginning (up to the depth of 1 mm) that decrease is rather small, almost nonexistent, for all the applied filler metals. Then, the sudden drop in hardness begins, down to about 400 HV, primarily for electrodes Inox + EDur and Castolin. For the third filler metal (UTP 670), the hardness drop occurs later (at depth of about 2 mm) and goes down to 500 HV. The decreasing trend then continues and remains at the similar rate for all the three filler metals, moving toward the base metal, where the hardness becomes uniform. These results are confirmed by the microstructure recordings, which show that the structure is coarse-grained, needle-like and dendritic, characterized by the high hardness [30]. By increasing the surface hardness, the teeth’s resistance to wear increases, as well as their service life [13]. The typical damages that appear on such surfaces are the appearance of initial holes and pitting on the teeth surfaces, which cause the irregular operation of gears. The appearance of pitting can be delayed by increasing the surface layers’ hardness [26]. In addition, it should be emphasized that the initial damage is the most likely to appear on the joint of the two electrode passes during the hard-facing. It is thus extremely important that the layers are well welded and joined to each other, so that no point could exist that could later act as a stress concentrator.

The gears’ regeneration resulted in numerous savings. Economic analysis of applied regeneration procedures assumes comparison of costs of the regenerated and newly manufactured gears. The regeneration price includes material and labor costs, as well as overheads. Compared to the price of the newly manufactured gears, considering the time of their service life, the advantages of hard-facing are multifold. The calculation of the techno-economic specific parameters, such as indicator of economic justification of regeneration, coefficient of the exploitation reliability and condition for the economic rationality of regeneration, are not given in this paper. In the previous research, by the authors of this paper, such a calculation was provided for a similar regeneration procedure, which confirms the aforementioned statements [1,13,32].

## 5. Conclusions

Research, results of which are reported in this paper, aimed to present the dependence of the physical–mechanical–metallurgical parameters of the gear teeth flanks, which are the technological inheritance of gears’ regeneration by hard-facing, on the type of the applied filler metal(s). The reparatory hard-facing of the gear teeth was executed by so-called “hard” electrodes, where the hard electrodes are made up of filler metals whose application results in such properties (mainly hardness) of surfaces that additional thermal-chemical treatment is not necessary.

The gears that were regenerated were not “pulled out” from use due to being damaged, but they were the newly produced and deliberately damaged gears. This ensured that all the tested gears were made of the same material (steel for cementation, 20MnCr5), belonged to one batch, all machined on the same machines by the same machining process. In this way, the results of experimental investigations reflected only the influence of application of the three different filler metals, while all other influences were eliminated. The three filler metals, used for regenerating hard-facing, were combination Inox 18/8/6 + EDur 600, Castolin 2 and UTP 670. The tested properties included geometrical accuracy, microstructure and microhardness. Evaluation of the executed regeneration procedures was done by comparing the mentioned parameters of the regenerated gears to the same parameters of the new ones.

Based on analysis of the obtained results from experimental investigations of the regenerated gears, the following conclusions were drawn:

- Geometrical characteristics, prescribed tolerances and deviations, as well as the quality of surfaces of the regenerated gears’ teeth flanks completely comply with prescribed and standardized values for the newly manufactured gears.
- The required mechanical properties, primarily the surface hardness, are easily obtained by the application of the adequate filler metal(s).
- The flanks of the regenerated gears possess a hardness almost equal to the hardness of the newly manufactured gears, provided that quality hard-facing and subsequent machining are ensured.
- Considering that electrodes, used for hard-facing, are characterized by the low heat input into the layers’ deposition zone, it is metallographically confirmed that the form of obtained surface microstructure is adequate.
- Analysis of the microhardness shows that the hard-faced layers executed by the “hard” filler metals possess a higher microhardness than the cemented and quenched base metal.

The general conclusion is that the best results of all the investigated characteristics are obtained for the teeth flanks of gears regenerated with the combination of filler metals Inox 18/8/6 + EDur 600. Thus, their application is recommended for reparatory hard-facing of damaged gears made of the steel for cementation, 20MnCr5.

## Figures and Tables

**Table 1:** Chemical composition of the 20MnCr5 steel [12].

Alloying Elements, %					
C	Si	Mn	P_max_	S_max_	Cr
0.17–0.22	0.15–0.40	1.1–1.4	0.035	0.035	1.0–1.3

**Table 2:** Characteristics of the tested gears [25].

Characteristics	Symbol	Value
Module	m	6 mm
Number of teeth	z	43
Base profile angle	α	20°
Tooth profile angle	β	0°
Pitch circle diameter	d_0_	258 mm
Profile correction	x_m_	0
Base circle diameter	d_b_	270 mm
Addendum circle diameter	d_f_	243.6 mm
Circular pitch	p	18.84 mm

**Table 3:** Notation and sizes of the used filler metals and applied current intensity.

No.	Notation			Manufacturer	D (mm)	I (A)
	DIN	AWS	DIN855			
1	E-6-UM-55G	-	EDur 600	Jesenice, Slovenia	2.50	70
2	-	-	Castolin 2	Castolin Eutectic, Switzerland	3.25	92
3	E-6-60-UM	-	UTP 670	UTP, Germany	3.25	90
4	E18.8.Mn6B20+	E307-15	Inox 18/8/6	Jesenice, Slovenia	2.50	70

**Table 4:** Chemical composition and mechanical properties of the applied filler metals.

No.		C	Si	Mn	Cr	Ni	Mo	Other	Hardness		R_m_	R_p02_
		%							HV	HRC	[MPa]	
1	EDur 600	0.5	2	-	9.5	-	-	-	-	54	-	-
2	Castolin 2			+	+	-	+		-	57–62	-	-
3	UTP 670	0.4	0.85	0.8	9.7	-	0.6	1.5 V	>600	-	-	-
4	Inox 18/8/6	0.12		7.0	19.0	9.0	-	-	-	-	590–690	>350

**Table 5:** Parameters of geometrical and kinematical accuracy of the controlled gears [12].

Controlled Parameter	Required Values	Gears Hard-Faced by		
		Inox 18/8/6 and EDur 600	Castolin 2	UTP 670
Base circle diameter d_b_ (mm)	270_−0.5_	269.82	269.77	269.77
Measure over 5 teeth W_5_ (mm)	83.32	83.12	83.18	83.11
Tooth profile tolerance T_ev_ (μm)	16	11	15	13
Tooth flank line deviation T_β_ (μm)	15	10	14	12
Radial tooth deviation T_r_ (μm)	78	33	60	52
Tooth surface machining quality	N7	N7 *	N7 *	N7 *
Surface hardness (HRC)	55–58	56.4	56.2	55.8

* R_a_ = 1.60 μm.

**Table 6:** The friction coefficients and the wear trace widths for samples hard-faced by different filler metals [12].

Disc		Block	Friction Coefficient	Wear Trace (mm)
Material	Hardness (HRC)	Filler Metal		
20MnCr5	55–58	Inox 18/8/6 + EDur 600	0.064	0.960
		Castolin 2	0.115	1.028
		UTP 670	0.090	0.955

**Table 7:** The surface hardness and macrohardness of the hard-faced and newly manufactured samples.

No.	Hardness HRC	Macrohardness	
		HV_30/15_	Corresponds to HRC
1	Inox 18/8/6 + EDur 600	57.5	63.3
2	Castolin 2	58	62.2
3	UTP 670	55.5	63.4

**Table 8:** Microhardness of the sample hard-faced with the Inox 18/8/6 + EDur 600 filler metals.

Distance from the surface (mm)	0.05	0.10	0.15	0.20	0.25	0.30	0.35	0.40	0.45	0.50	0.60	0.70	0.80
Hardness HV_01_	820	820	787	787	757	726	787	787	820	787	757	787	757
Corresponds to HRC	64.7	64.7	63.6	63.6	62.4	61.2	63.6	63.6	64.7	63.6	62.4	63.6	62.4
Distance from the surface (mm)	0.90	1.0	1.25	1.5	1.75	2.00	2.50	3.00	3.50	4.00	4.50	5.00	6.00
Hardness HV_01_	787	787	530	480	440	426	401	480	514	496	452	350	272
Corresponds to HRC	63.6	63.6	51.1	47.7	44.5	43.2	40.9	47.7	50.1	48.8	45.5	35.5	25.9

**Table 9:** Microhardness of the sample hard-faced with the Castolin 2 filler metal.

Distance from the surface (mm)	0.05	0.10	0.20	0.30	0.40	0.50	0.60	0.70	0.80	0.90	1.00	1.25
Hardness HV_01_	757	757	700	757	726	787	787	787	634	634	496	514
Corresponds to HRC	62.4	62.4	60.1	62.4	61.2	63.6	63.6	63.6	57.0	57.0	48.8	50.1
Distance from the surface (mm)	1.50	1.75	2.00	2.50	3.00	3.50	4.00	4.50	5.00	6.00	8.00	
Hardness HV_01_	514	473	401	452	401	440	401	440	350	350	350	
Corresponds to HRC	50.1	47.2	40.9	45.5	40.9	44.5	40.9	44.5	35.5	35.5	35.5	

**Table 10:** Microhardness of the sample hard-faced with the UTP 670 filler metal.

Distance from the surface (mm)	0.05	0.10	0.15	0.20	0.25	0.30	0.35	0.40	0.45	0.50	0.60	0.70	0.80
Hardness HV_01_	820	820	820	820	820	757	757	787	726	726	787	787	820
Corresponds to HRC	64.7	64.7	64.7	64.7	64.7	62.4	62.4	63.6	61.2	61.2	63.6	63.6	64.7
Distance from the surface (mm)	0.90	1.00	1.25	1.50	1.75	2.00	2.50	3.00	3.50	4.00	5.00	6.00	8.00
Hardness HV_01_	726	726	700	757	757	496	585	547	514	514	496	466	254
Corresponds to HRC	61.2	61.2	60.1	62.4	62.4	48.8	54.4	52.1	50.1	50.1	48.8	46.5	22.9

## Data Availability

The data presented in this study are available on request from the corresponding author.
